# Does one subgenome become dominant in the formation and evolution of a polyploid?

**DOI:** 10.1093/aob/mcac024

**Published:** 2022-03-15

**Authors:** Chunji Liu, You-Gan Wang

**Affiliations:** CSIRO Agriculture and Food, St Lucia, Queensland, Australia; Science and Engineering Facility, Queensland University of Technology, Brisbane, Queensland, Australia

**Keywords:** Polyploidy, polyploid evolution, subgenome dominance, whole-genome duplications, variable genome

## Abstract

**Background:**

Polyploids are common in flowering plants and they tend to have more expanded ranges of distributions than their diploid progenitors. Possible mechanisms underlying polyploid success have been intensively investigated. Previous studies showed that polyploidy generates novel changes and that subgenomes in allopolyploid species often differ in gene number, gene expression levels and levels of epigenetic alteration. It is widely believed that such differences are the results of conflicts among the subgenomes. These differences have been treated by some as subgenome dominance, and it is claimed that the magnitude of subgenome dominance increases in polyploid evolution.

**Scope:**

In addition to changes which occurred during evolution, differences between subgenomes of a polyploid species may also be affected by differences between the diploid donors and changes which occurred during polyploidization. The variable genome components in many plant species are extensive, which would result in exaggerated differences between a subgenome and its progenitor when a single genotype or a small number of genotypes are used to represent a polyploid or its donors. When artificially resynthesized polyploids are used as surrogates for newly formed genotypes which have not been exposed to evolutionary selection, differences between diploid genotypes available today and those involved in the formation of the natural polyploid genotypes must also be considered.

**Conclusions:**

Contrary to the now widely held views that subgenome biases in polyploids are the results of conflicts among the subgenomes and that one of the parental subgenomes generally retains more genes which are more highly expressed, available results show that subgenome biases mainly reflect legacy from the progenitors and that they can be detected before the completion of polyploidization events. Further, there is no convincing evidence that the magnitudes of subgenome biases have significantly changed during evolution for any of the allopolyploid species assessed.

## INTRODUCTION

Polyploidy is a process of forming new species by enveloping two or more genomes within a single nucleus. It is now widely believed that whole-genome duplication (WGD) has been a recurrent process in the evolution of flowering plants. Available results show that there are several WGDs in the early lineages leading to angiosperms (Alix *et al.*, 2017), and signals for WGDs have been reported among diverse groups of angiosperms (Vision *et al.*, 2000; Bowers *et al.*, 2003; Jiao *et al.*, 2011). Lineage-specific WGDs are also common. For instance, signals for more than one WGD have been detected before the arabidopsis*–Brassiceae* split (Bowers *et al.*, 2003), and a *Brassiceae* lineage-specific whole-genome triplication has been reported (Lysak *et al.*, 2005; Wang *et al.*, 2011; Liu *et al.*, 2014). Similarly, signals for a WGD before the divergence of the major cereals from one another were detected (Paterson *et al.*, 2004; Tang *et al.*, 2010), and WGD has also occurred in rice since its divergence from other cereals (Yu *et al.*, 2005). As a result of these WGDs, genuine diploid flowering plant species may not exist. All flowering plants, including *Arabidopsis thaliana*, are believed to be ancient polyploids (Blanc *et al.*, 2000; Vision *et al.*, 2000).

Polyploids can be divided into autopolyploids and allopolyploids. The former contains multiple copies of the same genome in a single nucleus, and the latter contains two or more different (sub-)genomes within a single nucleus. As they contain different subgenomes, it is easier to identify which sequence belongs to which subgenome in an allopolyploid. Many important crop species, including bread wheat (*Triticum aestivum*), durum wheat (*T. durum*), cotton (*Gossypium arboretum*) and oilseed rape (*Brassica napus*), are typical allopolyploids. Compared with their progenitor species, polyploids tend to have better ability to colonize new environmental niches and have expanded ranges of distribution (Hegarty and Hiscock, 2008; te Beest *et al.*, 2012). The positive correlation between ploidy level and success of a species is also exemplified by domesticated wheat (Dubcovsky and Dvorak, 2007). In almost all areas where domesticated einkorn (*T. boeoticum*, diploid) and domesticated emmer (*T. dicoccum,* tetraploid) were cultivated, it was the domesticated emmer that became dominant. Compared with both the diploid and tetraploid wheats, the hexaploid common wheat (*T. aestivum*) expanded much further and it is grown from Norway and Russia at 65°N to Argentina at 45°S (Dvorak *et al.*, 1998). Not surprisingly, the topic of understanding why polyploid species are so successful in evolution has attracted a great deal of attention in the scientific community, and possible underlying mechanisms have been proposed (Soltis and Soltis, 2000; Hegarty and Hiscock, 2008; Alix *et al.*, 2017; Van de Peer *et al.*, 2021). For instance, Dubcovsky and Dvorak (2007) proposed that polyploid genomes have better plasticity, which is a key factor in their success during evolution and selection. Some believe that newly generated variations in polyploids may provide them with improved evolutionary potential and adaptive capabilities (Soltis *et al.*, 2014) and that regulation of meiotic recombination is closely related to polyploid success (Pelé *et al.*, 2018). Van de Peer *et al.* (2021) hypothesized that stress response in general is an important and even a determining factor in the establishment and success of polyploids.

Before generating and analysing large quantities of genome sequences became practical, studies on changes between subgenomes during and following polyploidization were conducted using small numbers of genes or sequences. Results from some of these studies showed that allopolyploidy is accompanied by rapid and non-random gene loss as well as silencing and activation of DNA sequences (Feldman *et al.*, 1997; Ozkan *et al.*, 2001; Shaked *et al.*, 2001; Kashkush *et al.*, 2002; Gaeta *et al.*, 2007; Jiao *et al.*, 2018). It was believed that such rapid changes should be expected as previously separate genomes must adjust and coexist with one another in a single nucleus of a polyploid genotype. However, such changes do not seem to be universal as they were not detected in the formation of some allopolyploid species including cotton (Liu *et al.*, 2001; Page *et al.*, 2016) and Arabidopsis (Wang *et al.*, 2006; del Pozo and Ramirez-Parra, 2015). In any case, due to the limited numbers of genes or sequences assessed, results from such studies should not be extrapolated to represent the existence of subgenome dominance as the numbers of assessed genes or sequences were too small to alter the overall difference between genomes or subgenomes.

**Fig. 1.**
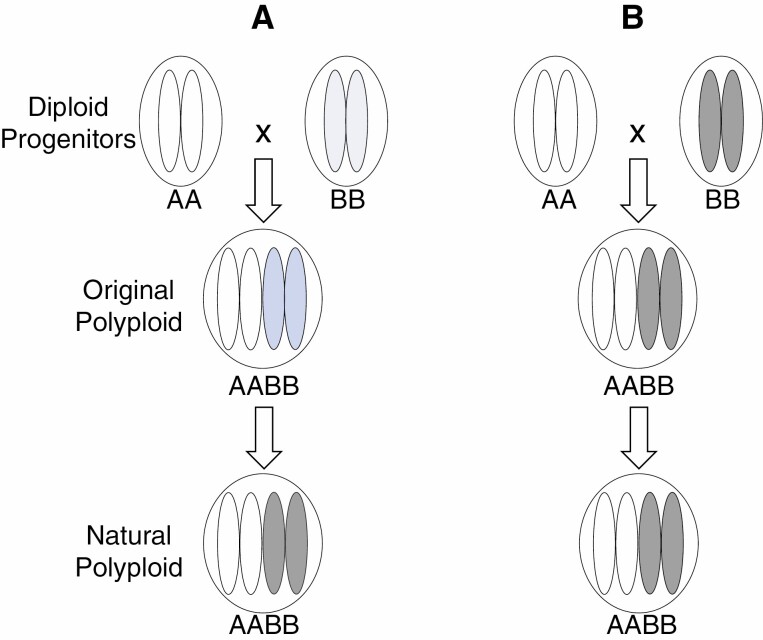
A diagram illustrating two possible scenarios of subgenome biases in polyploid formation and evolution as discussed herein, one being that the degree of increases of subgenome biases over time (A) and the other that subgenome biases mainly reflect differences between diploid donors (B). Each of the four oval shapes contained in each of the nuclei (circles) represents a haploid gamete produced by a diploid individual. Darker colours represent higher degrees of subgenome biases.

Since analysing large numbers of sequences became feasible, subgenome biases in genome size, contents of transposable elements, gene number, gene expression level or epigenetic alteration have been reported for many allopolyploid species (e.g. Hendrix and Stewart, 2005; Flagel and Wendel, 2010; Guo and Han, 2014; A. Li *et al.*, 2014; Edger *et al.*, 2017; Ding and Chen, 2018; VanBuren *et al.*, 2020). Some hypothesized that genetic incompatibilities among subgenomes probably contribute to these differences, resulting in one of the parental subgenomes in a polyploid generally retaining more genes which are more highly expressed. This phenomenon is termed subgenome dominance, and some believe that the magnitude of subgenome dominance increases over time (Fig. 1A) (Edger *et al.*, 2017; Alger and Edger, 2020).

Recent studies reveal that an individual genotype does not possess all the genes of a species. Genes present in all individuals of a species are termed the core genome component, and those not present in all individuals are termed the dispensable (DGC) or variable genome component (VGC) (Wing, 2015). Available analyses suggest that VGC accounts for at least 20 % of the genomes in soybean (*Glycine soja*) (Y.H. Li *et al.*, 2014) and *Brassica oleracea* (Golicz *et al.*, 2016), >36 % in bread wheat (*T. aestivum*) (Liu *et al.*, 2016; Montenegro *et al.*, 2017; Walkowiak *et al.*, 2020), 38 % in barley (Ma *et al.*, 2019; Jayakodi *et al.*, 2020), 50 % in maize (*Zea mays*) (Hirsch *et al.*, 2014; Jin *et al.*, 2016) and 43 % in rice (*Oryza sativa* L.) (Sun *et al.*, 2017). Importantly, these large estimates of VGC were obtained by evaluating from a few to a few dozen genotypes in each species and the estimated VGC will become larger when additional genotypes are included in such assessments.

The existence of VGC means that some of the practices routinely followed inevitably lead to biases in studying differences between subgenomes. One of the examples is the practice of aligning genomic or RNA sequences obtained in a study against the reference genome of the species of concern (F. Li *et al.*, 2014; Jiao *et al.*, 2018; Bird *et al.*, 2021). While there are good reasons for such a routine practice, it would result in the loss of all those sequences not present in the reference genome. Similarly, the existence of VGC means that differences between a subgenome and its donor would probably be exaggerated when sequences from only a single genotype or a small number of genotypes are used to represent a polyploid or one of its progenitors.

## FACTORS AFFECTING ESTIMATION OF SUBGENOME BIASES

**Fig. 2.**
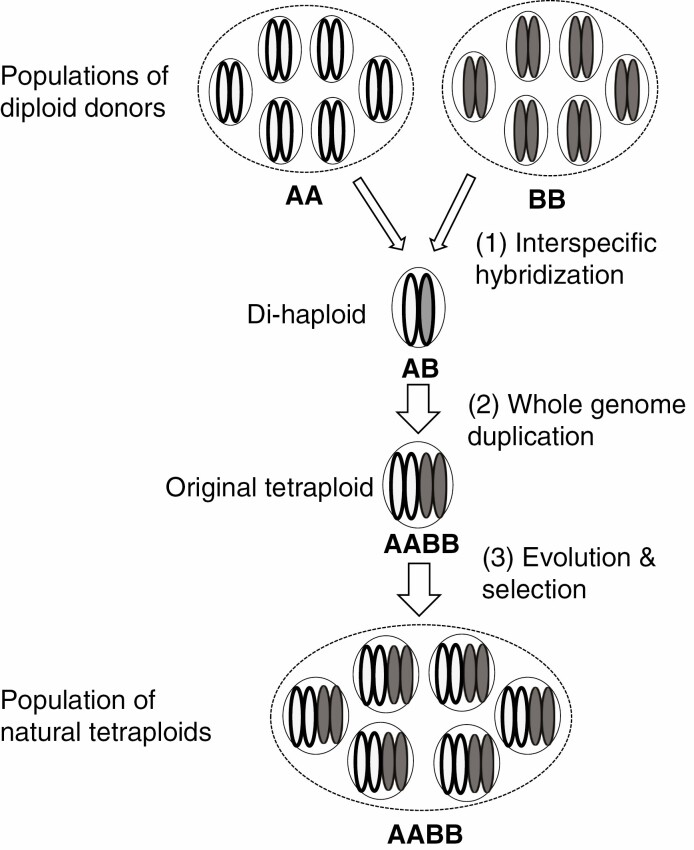
A diagram showing the major steps, thus factors that need to be considered, in studying subgenome biases in the formation and evolution of allopolyploids. Six individuals are used to represent each of the populations for both the diploid donors and the natural polyploids derived from them.

As VGC can be substantial in a species, some differences between a subgenome and its progenitor should be expected even if true differences between them do not exist. With this understanding, we re-examined available publications on subgenome dominance and asymmetric evolution in polyploid species. As discussed below, our examinations found no strong evidence showing either that one subgenome in a polyploid retained more genes in polyploid formation or that the magnitudes of subgenome biases have changed much during allopolyploid formation and evolution (Fig. 1B). To facilitate the explanation of our arguments, we would like to emphasize the key stages in polyploid formation and evolution that should be considered in evaluating subgenome biases (Fig. 2). It is of note that allopolyploids may also derive from the union of unreduced gametes (Ramsey and Schemske, 1998), in which the di-haploid hybrid stage does not exist. However, all artificial allopolyploids used in previous publications on subgenome biases are generated from interspecific hybridization (e.g. Feldman *et al.*, 1997; Liu *et al.*, 2001; Gaeta *et al.*, 2007; Guo and Han, 2014; Edger *et al.*, 2017; El Baidouri *et al.*, 2017; Bird *et al.*, 2021). For these reasons, we do not intend to use the diagram to reflect the genuine relationships among the different classes of genotypes. Rather, it is intended to highlight key comparisons that need to be considered in estimating contributions of polyploid formation and evolution to subgenome biases. They include the followings:

### Differences between progenitors and allopolyploids derived from them

Available results show beyond any doubt that differences between the subgenomes of a polyploid probably reflect, at least to a large degree, those between their donors. This is the case in all studies where differences between progenitors and allopolyploids derived from them were compared. For example, Bird *et al.* (2021) analysed gene expression in six resynthesized *B. napus* allopolyploid lines and found that subgenome expression bias is consistent over the first ten generations. They showed that gene expression favoured the same subgenome in all resynthesized allopolyploid lines and generations, and that the majority of the biased gene pairs showed the same dominance patterns across all lines and in an *in silico* hybrid of the parents. Similarly, the relative gene diversity among the three subgenomes of the various allopolyploid species of *Brassica* correlate well with those among their diploid donors (Ye *et al.*, 2021). Results from previous studies also show that the large differences in both genome sizes and the contents of transposable elements between the two subgenomes of tetraploid cotton (Zhang *et al.*, 2015) also exist between the genomes of the two putative donor species (Hendrix and Stewart, 2005; F. Li *et al.*, 2014). These results provide further evidence showing the importance of parental legacies in alloploid formation and evolution (Fig. 1B) as described by Gottlieb and colleagues (Roose and Gottlieb, 1980; Gottlieb, 2003; Buggs *et al.*, 2014).

Clearly, differences between a subgenome and its donor must be considered when assessing subgenome biases. It is also important to remember that only a single individual, not the whole species, of the donor species was involved in forming a polyploid genotype. Thus, the possible impact of VGC also needs to be considered when the exact individuals involved in forming a polyploid of concern are unknown.

### Difference between a transit F_1_ hybrid and the allopolyploid from it

Several studies compared subgenome biases between allopolyploids and their respective F_1_ hybrids. Without exception, results from these studies all show that F_1_ hybrids and the polyploids derived from them always share the same ‘dominant’ subgenomes with similar magnitudes of subgenome biases. Examples include the studies on wheat (Ozkan *et al.*, 2001; Ye *et al.*, 2021), *Solanum lycopersicum* or monkeyflower (Edger *et al.*, 2017) and *B. napus* (Bird *et al.*, 2021). Results from these studies show clearly that subgenome biases are not the products of polyploidization.

### Differences between newly synthesized and natural polyploid genotypes

Importantly, the existence of subgenome dominance cannot be determined by analysing natural polyploid genotypes solely, although such results have been used as evidence of subgenome dominance in many studies (Schnable *et al.*, 2011; Feldman and Levy, 2012; Guo and Han, 2014; El Baidouri *et al.*, 2017; Edger *et al.*, 2019; Chen *et al.*, 2021). As is well known, several steps separate natural polyploid genotypes from their diploid progenitors (Fig. 2). Artificial polyploids have been generated and used as surrogates for the ‘original’ polyploids in some studies. This practice assumes that, like the original polyploids, artificial polyploids have hardly been exposed to evolution and selection. Without exception, all published research shows that these two different types of polyploids always share the same ‘dominant’ subgenome with similar magnitudes of subgenome biases. For example, comparing genome-wide gene expression changes between artificial and natural allohexaploid wheat genotypes found that subgenome biases favour the same subgenome between these two types of allopolyploid genotypes (Chagué *et al*., 2010). An analys of five different allotetraploid cotton species found that homoeologue expression biases favour the D subgenome in each of them (Flagel and Wendel, 2010). Ye *et al.* (2021) compared differences between natural and artificial allopolyploid genotypes for six different allopolyploid species, namely cotton, wheat, arabidopsis and three different allotetraploid *Brassica* species (*B. napus*, *B. carinata* and *B. juncea*). These assessments also found no significant differences in the levels of subgenome biases between the artificial and natural allopolyploid genotypes for any of these species.

It is of note that some of the publications cited above claimed that subgenome dominance increases in magnitude over time. For instance, Edger *et al.* (2017) compared gene expression differences among a di-haploid hybrid, an artificial allopolyploid and a natural genotype of *Minulus peregrinu*, and claimed that subgenome dominance at the gene expression level increased over subsequent generations in this polyploid species. This publication was also cited by Alger and Edger (2020) when proposing that subgenome dominance increases over time. Similar to results from other species described above, the percentages of homoeologues with biased expression were found to be higher in the same subgenome from the two different polyploids and the transit F_1_ hybrid. Further, some reported differences may not be statistically significant. Biased expression of homoeologues between the two assessed subgenomes among the three genotypes is one of them (Box 1). Clearly, these results do not support that subgenome biases at the gene expression level increased over subsequent generations in this species. The subtle differences detected in the above study can be caused by any of the following factors: (1) differences between the diploid donors; (2) possible impact of VGC as only a single genotype was used for each of the three different types of materials; (3) even for those allopolyploid species for which their exact progenitor species are known, the genotypes of the progenitor species available today are different from those involved in the formation of the initial polyploid genotypes from which the currently available natural polyploid genotypes were derived. Like the polyploid derived from them, these modern-day diploid progenitor genotypes are also the product of evolution and selection; (4) it is well known that both gene expression and epigenetic alteration are tissue specific. Results from previous studies showed unambiguously that, depending on tissues or time tested, ‘dominant’ subgenomes may vary for a given allopolyploid species (A. Li *et al.*, 2014; Hu *et al.*, 2015; Wang *et al.*, 2016; Colle *et al.*, 2019; Kryvokhyzha *et al.*, 2019). The concept of ‘a spatiotemporal-specific subgenome’ was used to describe such inconsistent results in a recent publication arguing for subgenome dominance (Alger and Edger, 2020). However, the tissue- and time-specific nature of such results means that such results are not strong evidence for either subgenome dominance or asymmetric evolution.

Importantly, we are not questioning that polyploidy generates novelties, a phenomenon which has been known for a long time (Levin, 1983). Profiles of dimeric enzymes (Roose and Gottlieb, 1976; Liu and Gale, 1989) serve as a good example for such novelties. When two or more different genomes come together in an allopolyploid, novel enzymes that do not exist in any of the progenitor genotypes would be produced from inter-locus interactions (Soltis *et al.*, 2014). However, such novelties do not favour a given subgenome and thus should not alter the overall differences among them in a polyploid.

### Results on subgenome dominance from studying ‘ancient polyploids’

With the belief that WGDs or polyploidization have occurred in the evolution of most, if not all, flowering plant species (e.g. Bowers *et al.*, 2003; Lysak *et al.*, 2005; Jiao *et al.*, 2011; Wang *et al.*, 2011), results from some typical diploid species have also been used in arguing for subgenome dominance or asymmetric evolution. For example, when reporting the genome sequence of the now typical diploid *B. oleracea*, Liu *et al.* (2014) claimed evidence of asymmetrical evolution. Another example is the report by Zhao *et al.* (2017) who analysed difference between maize and soybean in many features including gene expression, rates of transposable element accumulation, levels of small interfering RNAs and DNA methylation around genes, and rates of gene loss. Based on these differences, the authors speculated that, compared with those of maize, the two subgenomes of soybean were more distinct prior to the allotetraploidization event. Clearly, both maize and soybean were treated in these studies as the products of ancient allopolyploidization. However, even assuming that these species are indeed the products of ancient polyploidization, the results described in these publications still cannot be treated as evidence for subgenome dominance or asymmetric evolution. This is because the original states of such ‘polyploid’ species or their progenitors are not known. These unknowns make it impossible to work out how much of the differences between the ‘subgenomes’ of such ‘polyploids’ reflect those between their donor species, or what proportions of the ‘changes’ have been accumulated during subsequent evolution. Thus, speculation derived from studying such ancient polyploid species on either subgenome dominance or asymmetric evolution should only be treated as such.

## CONCLUSION

We do not dispute that rapid and non-random gene loss, silencing and activation of DNA sequences can occur following allopolyploidization in at least some species. We also do not question that novelties occur in allopolyploid formation and evolution. However, strong evidence showing either the existence of subgenome dominance or the increase of its magnitude in polyploid formation and evolution does not seem to exist. Available results show that differences between subgenomes of a polyploid reflect, at least to a large degree, the legacy from its diploid progenitors. The existence of VGC and the differences between modern day donors and those involved in forming the polyploid species under investigation are two additional factors that must be considered in studying subgenome biases. As pointed out by Dubcovsky and Dvorak (2007), genome plasticity due to their polyploid nature can be the key factor underlying the success of such species. There are no reasons to rule out the possibility that the success of a polyploid can also be influenced by a small number of genes and that such genes may differ for the same polyploid species among different environments.

Box 1. **The significance test**

The null hypothesis is *p*_1_ = *p*_2_= *p*_3_, or d_1_ = *p*_2_ − *p*_1_ = 0 and *d*_2_ = *p*_3_ − *p*_2_ = 0. The approximate *χ*^2^ test statistic (2 degrees of freedom) is

$$\begin{array}{l} d_{1}\;d_{2}\sum_{}^{-1}\left(\begin{array}{l} d_{1}\\ d_{2} \end{array}\right), \end{array}$$

where Σ is the covariance of the estimates (*d*_1_,*d*_2_), and

$$\begin{array}{l} \Sigma =\left(\begin{array}{cc} \frac{p_{1}(1-p_{1})}{n_{1}}+\frac{p_{2}(1-p_{2})}{n_{2}} & \frac{p_{2}(1-p_{2})}{n_{2}}\\ \frac{p_{2}(1-p_{2})}{n_{2}} & \frac{p_{3}(1-p_{3})}{n_{3}}+\frac{p_{2}(1-p_{2})}{n_{2}} \end{array}\right) \end{array}$$

The numbers of homoeologs with biased expression for the three genotypes obtained by Edger *et al.* (2017) were *n*_1_ = 388 + 336 = 724, *n*_2_ = 329 + 348 = 677, and *n*_3_ = 341 + 380 = 721, respectively: and the gene ratios between the two subgenomes for the three genotypes were 52% (*p*_1_), 51% (*p*_2_), and 53% (*p*3), respectively. The *p*-value based on these numbers is 0.179, indicating that there are no significant differences among the three ratios.
